# Berberine Enhances the Antibacterial Activity of Selected Antibiotics against Coagulase-Negative Staphylococcus Strains *in Vitro*

**DOI:** 10.3390/molecules19056583

**Published:** 2014-05-22

**Authors:** Robert D. Wojtyczka, Arkadiusz Dziedzic, Małgorzata Kępa, Robert Kubina, Agata Kabała-Dzik, Tomasz Mularz, Danuta Idzik

**Affiliations:** 1 Department and Institute of Microbiology and Virology, School of Pharmacy and Division of Laboratory Medicine in Sosnowiec, Medical University of Silesia, Katowice, ul. Jagiellońska 4, 41-200 Sosnowiec, Poland; E-Mails: mkepa@sum.edu.pl (M.K.); tomekmularz@aol.com (T.M.); didzik@sum.edu.pl (D.I.); 2 Department of Conservative Dentistry with Endodontics, Medical University of Silesia, Katowice, Pl. Akademicki 17, 41-902 Bytom, Poland; E-Mail: adziedzic@sum.edu.pl; 3 Department and Institute of Pathology, School of Pharmacy and Division of Laboratory Medicine in Sosnowiec, Medical University of Silesia, Katowice, ul. Ostrogórska 30, 41-200 Sosnowiec, Poland; E-Mails: rkubina@sum.edu.pl (R.K.); adzik@sum.edu.pl (A.K.-D.)

**Keywords:** antimicrobial activity, *coagulase-negative staphylococci* (CoNS), berberine

## Abstract

Synergistic interactions between commonly used antibiotics and natural bioactive compounds may exhibit therapeutic benefits in a clinical setting. Berberine, an isoquinoline-type alkaloid isolated from many kinds of medicinal plants, has proven efficacy against a broad spectrum of microorganisms. The aim of the presented work was to assess the antibacterial activity of berberine chloride in light of the effect exerted by common antibiotics on fourteen reference strains of *Staphylococccus* spp., and to evaluate the magnitude of interactions of berberine with these antistaphylococcal antibiotics. In our study minimum inhibitory concentrations (MIC) of berberine chloride against CoNS ranged from 16 to 512 µg/mL. The most noticeable effects were observed for *S. haemolyticus* ATCC 29970, *S. epidermidis* ATCC 12228, *S. capitis* subsp*. capitis* ATCC 35661, *S. galinarium* ATCC 700401, *S. hominis* subsp*. hominis* ATCC 27844, *S. intermedius* ATCC 29663 and *S. lugdunensis* ATCC 49576. The most significant synergistic effect was noticed for berberine in combination with linezolid, cefoxitin and erythromycin. The synergy between berberine and antibiotics demonstrates the potential application of compound combinations as an efficient, novel therapeutic tool for antibiotic-resistant bacterial infections.

## 1. Introduction

The widespread abuse of antibiotics for the treatment of bacterial infections has led to the emergence and spread of drug resistant strains. The rise in the rate of infections from multi-drug resistant (MDR) bacteria is recognized worldwide as a major health crisis. A recent joint technical report of the European Centre for Disease Prevention and Control (ECDC) and the European Medicines Agency (EMA) in collaboration with Action on Antibiotic Resistance (ReAct) estimated that at least 25,000 patients die each year in the EU from infections due to multidrug resistant bacteria [1]. Antibiotic resistant staphylococci strains are the major public health concern since the bacteria can circulate in the environment without difficulty. *Coagulase-negative staphylococci* (CoNS) colonize different parts of the human skin and mucous membranes. Notably, every species of CoNS that has been characterized as a resident in humans (*S. epidermidis*, *S. capitis*, *S. cohnii*, *S. haemolyticus*, *S. hominis*, *S. lugdunensis*, *S. saccharolyticus*, *S. saprophyticus*, *S.warneri*), may also be responsible for nosocomial infections, particularly in immunocompromised patients. What is more, CoNS have become the leading cause of infections related to medical devices such as vascular catheters, prosthetic joints and artificial heart valves [2,3].

Plants are known to produce a variety of compounds as defenses against a wide range of microorganisms. Berberine is an isoquinoline-type alkaloid isolated from many kinds of medicinal plants such as *Berberis aristata, Berberis aquifolium, Berberis vulgaris, Coptis chinensis*, *Coptis japonica*, *Hydrastis canadensis*, *Phellodendron amurense,* *Phellodendron chinense schneid* and other species [4,5,6]. Berberine has antidiabetic, antidiarrhoeal, antimicrobial, immuno-stimulating, hypotensive and anti-inflammatory properties [4,5,6,7,8]. To evaluate its antibacterial activity, most studies have focused on the bacteriostatic and/or bactericidal activities of berberine toward different bacterial species [9,10]. It has been reported that berberine has weak activity against Gram-negative bacteria and is more active against Gram-positive bacteria including *S. aureus* and *S. epidermidis* [7]. Moreover, the toxicity and mutagenicity of berberine to human cells were relatively low in both *in vitro* and *in vivo* experiments. [8,11,12]. To our knowledge, isoquinoline-type alkaloids have not yet been investigated in the context of their possible synergistic effects with commonly used antibiotics against CoNS strains.

It has been shown that pharmacological treatment by some phytochemicals is an inexpensive, readily applicable approach in the chemotherapy and management of various infections [5,6,8]. Due to the multi-drug resistance problem the use of combinations of antibiotics with the different mechanisms of action is often necessary for the treatment of severe staphylococcal infections. The augmented action of antibiotics along with natural substances may have positive synergistic effects toward specific, drug resistant microorganisms which are difficult to eradicate, particularly in hospital settings. In this paper, we explored *in vitro* antimicrobial activity of berberine in combination with 10 different antibiotics (penicillin—P, erythromycin—E, clindamycin—DA, cefoxitin—FOX, ciprofloxacin—CIP, tobramycin—TOB, chloramphenicol—C, linezolid—LIN, tetracycline—TE, trimethoprim with sulfamethoxazole—SXT) against 14 reference strains of *Staphylococccus* spp. using minimum inhibitory concentration (MIC) and time-kill assays. All these antibiotics have been reported as anti-staphylococcal drugs with different target points.

## 2. Results and Discussion

### 2.1. Activity of the Berberine

The antibacterial activity of berberine against the tested CoNS strains varied, with MIC values that ranged from 16 to 512 µg/mL (median 126 µg/mL, Table 1). The highest MIC values were 16 µg/mL for *S. capitis* subsp. *capitis* and 32 µg/ mL for *S. epidermidis* ATCC 12228. In case of 9 examined strains MIC values were within the range from 64 µg/mL to 128 µg/mL. The lowest MIC values were observed for *S. warneri* ATCC 49454 strain (512 µg/mL), *S. saprophyticus* ATCC 15303 (512 µg/mL) and *S.* *haemolyticus* ATCC 29970 strain (256 µg/mL). Comparison of non-biofilm forming strain of *S. epidermidis* (ATCC 12228) and biofilm-forming strain (ATCC 35983), showed that the first one mentioned strain was four times less susceptible to berberine, with MIC values obtained as 32 µg/mL and 128 µg/mL respectively.

The berberine used in this study significantly inhibited the growth of all examined bacterial strains.

**Table 1:** MIC (expressed in µg/mL) of berberine against fourteen CoNS strains.

Bacterial strain	MIC (µg/mL)
*S. epidermidis* ATCC 12228	32
*S. epidermidis* ATCC 35983	128
S. *haemolyticus* ATCC 29970	256
*S. hominis* subsp*. hominis* ATCC27844	64
*S. warneri* ATCC 49454	512
*S. saprophyticus* ATTC 15303	512
*S. capitis* subsp*. capitis* ATCC 35661	16
*S. intermedius* ATCC29663	64
*S. lentus* ATCC 700403	64
*S. lugdunensis* ATCC 49576	64
*S. simulans* ATCC 27851	128
*S. galinarium* ATCC 700401	128
*S. sciuri* ATCC 29060	128
*S. xylosus* ATCC 700404	128

### 2.2. Time-Kill Assay

**Figure 1:**
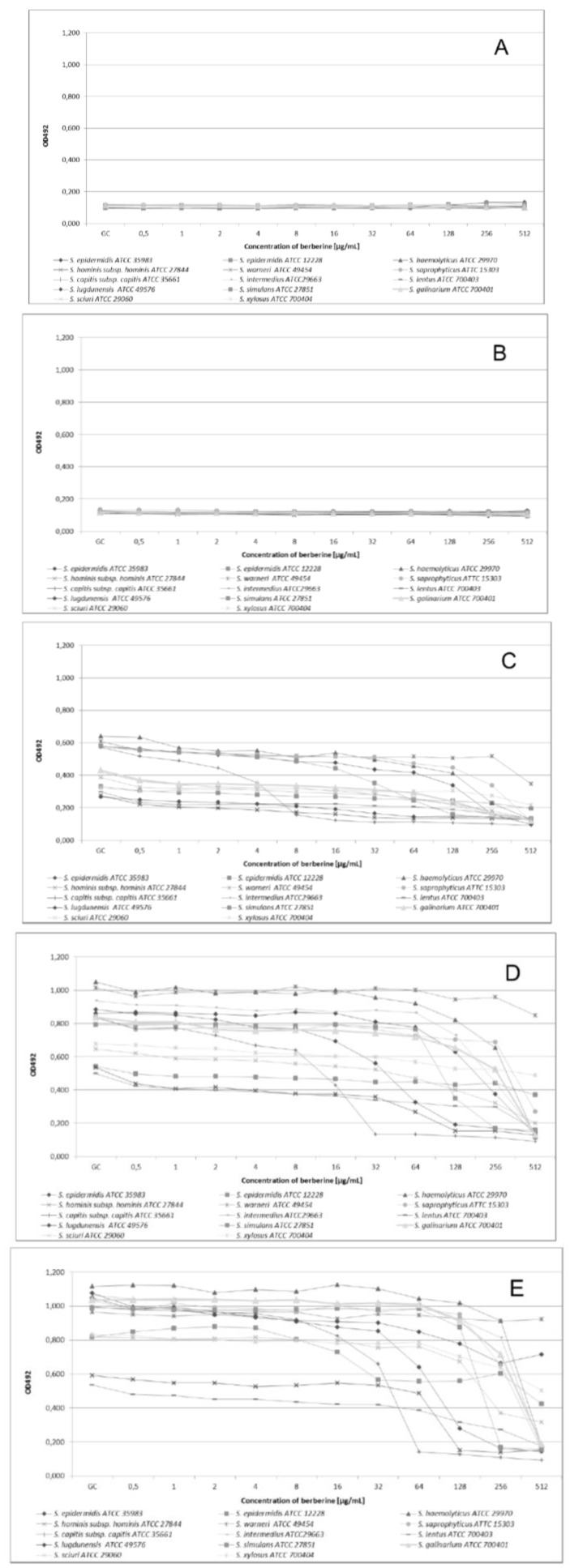
Growth kinetics of *Staphylococcus* strains in the presence of different berberine concentrations after 0 h of incubation (**A**); after 2 h of incubation (**B**); after 6 h of incubation (**C**); after 12 h of incubation (**D**) and after 24 h of incubation (**E**).

After 2 h of incubation no growth of the tested strains was detected, both with and without berberine addition to the medium (Figure 1B). There were no differences between culture growth when comparing time-kill curves for different experiment starting points (Figure 1A). After 6 h of incubation we noted for all CoNS strains a substantial decrease of the number of microorganisms (evidenced by OD value changes) when compared to growth control (GC, Figure 1C). In the 12th h of the study (Figure 1D) we observed an essential decrease of the number of microorganisms related to the berberine concentration within the range from 8 µg/mL to 512 µg/m. After 24 h of experiment (Figure 1E) within the range of berberine concentrations from 32 µg/mL to 512 µg/mL, a total growth inhibition was recorded for some strains, and no change of OD values was observed. The data obtained for *S. warneri* ATCC49454 strain showed that for all tested berberine concentrations, only small reduction of bacteria growth was observed, which may indicate an obvious strain resistance to berberine. In the case of the biofilm-forming strain *S. epidermidis* ATCC35983, a significant reduction of microorganism growth was observed for the first 12 h, but after 24 h of incubation we detected a subsequent increase of the OD value apparently related to the biofilm formation phenomenon. However, even for this non-biofilm forming strain a reduction of the number of microorganisms, expressed as an OD change, was noticeable.

The ANOVA indicated that the growth kinetics of all staphylococcal strains was significantly affected by berberine incubation time (*p* < 0.001), type of strains (*p* < 0.001), interaction between strain and incubation time and concentration. The interaction between these factors was also significant (*p* < 0.001). The incubation time effect (68.35%), type of strain (7.43%), interaction between type of strain and incubation time (6.93%) and concentration (6.03%) explained most of variance (Table 2).

**Table 2:** Multivariate analysis of variance by three-way ANOVA of CoNS susceptibility to berberine.

Factors	df	Sum of Squares	Mean Squares	F	% of Variance	*p*
strain (S)	13	26.8035	2.0618	6016	7.43	<0.001
time (T)	4	246.5799	61.6450	179878	68.35	<0.001
concentration (C)	11	21.7349	1.9759	5766	6.03	<0.001
SxT	52	24.9890	0.4806	1402	6.93	<0.001
SxC	143	8.8737	0.0621	181	2.46	<0.001
TxC	44	17.4305	0.3961	1156	4.83	<0.001
SxTxC	572	13.4642	0.0235	69	3.73	<0.001

### 2.3. Effects of Combinations of Berberine and Antibiotics against CoNS

A combined effect of berberine and antibiotics was observed for seven out of 14 tested CoNS strains. A significant increase of the growth inhibition zone by more than 5 mm was observed around antibiotic discs after the addition of ¼ MIC berberine into the MHA medium (Wilcoxon Signed-Rank Test *p* ≤ 0.05, Table 3). We did not observe any antagonistic interaction for any antibiotic-berberine combination studied.

The biggest differences in growth inhibition zone were observed for six of the investigated CoNS strains. An increase of the growth inhibition zone ranging from 9 to 18 mm was observed for the *S. haemolyticus* ATCC29970 strain following the addition of ¼ MIC berberine into the MHA medium. The greatest differences of growth inhibition zone size were observed for TE (increase from 30 to 48 mm), LIN (increase from 31 to 45 mm), TOB (increase from 26 to 40 mm), C (increase from 27 to 41 mm), FOX (increase from 33 to 47 mm), P (increase from 33 to 46 mm) and DA (increase from 26 to 37 mm). For the above antibiotics we observed a substantial enhancement of the growth inhibition zone from 11 to 18 mm. 

In the case of the *S. epidermidis* ATCC12228 strain, the increase of the growth inhibition zone after addition of ¼ MIC berberine into MHA medium ranged from 2 to 14 mm (excluding CIP). The biggest differences in the size of the inhibited growth zones were seen for E (increase from 27 to 41 mm), TOB (increase from 21 to 47 mm), C (increase from 28 to 39 mm), LIN (increase from 34 to 44 mm), P (increase from 12 to 22 mm), TE (increase from 1 to 9 mm) and for DA (increase from 27 to 35 mm). For these antibiotics, the growth inhibition zone increase ranged between 8 to 14 mm.

**Table 3:** The combined antimicrobial effect of antibiotics and berberine towards the reference CoNS staphylococci (mean inhibition growth zones in mm. Values are considered significant at *p* ≤ 0.05).

Strain	P	P	E	E	DA	DA	FOX	FOX	CIP	CIP	TOB	TOB	C	C	LIN	LIN	TE	TE	SXT	SXT
		+be		+be		+be		+be		+be		+be		+be		+be		+be		+be
*S. epidermidis* ATCC35983	0	0	0	0	0	0	12 ± 2	18 ± 2	30 ± 2	32 ± 2	17	18 ± 2	23 ± 2	26 ± 2	30 ± 3	33 ± 1	27 ± 1	29 ± 1	29 ± 1	31 ± 1
*S. epidermidis* ATCC12228	12 ± 2	22 ±1	27 ± 1	41 ± 1	27 ± 1	35 ± 1	34 ± 3	36 ± 2	28 ± 1	27 ± 1	27 ± 2	41 ± 1	28 ± 1	39 ± 1	33 ± 2	44 ± 2	0	9 ± 3	23 ± 2	23 ± 2
*S. haitalicolyticus* ATCC29970	33 ± 1	46 ± 2	26 ± 2	35 ± 3	26 ± 1	37 ± 1	33 ± 1	47 ± 3	30 ± 1	39 ± 1	26 ± 1	40 ± 1	27 ± 1	41 ± 3	31 ± 2	45 ± 1	30 ± 1	48 ± 2	22	40
*S. hominis* subsp*. hominis* ATCC27844	34 ± 2	38 ± 1	34 ± 2	40	29 ± 1	34 ± 2	32	41 ± 1	31 ± 2	33 ± 1	27 ± 1	30 ± 2	30 ± 2	35 ± 1	34 ± 3	45 ± 3	11 ± 1	12 ± 2	32	33 ± 1
*S. warneri* ATCC49454	31 ± 2	31 ± 3	24 ± 2	26 ± 1	28 ± 2	28	32	37 ± 1	27 ± 1	30 ± 2	25 ± 1	26	26 ± 2	29 ± 3	31 ± 2	33 ± 2	31 ± 3	33 ± 1	31 ± 1	33 ± 2
*S. saprophyticus* ATTC15303	27 ± 1	29	29 ± 1	32 ± 1	28	33 ± 1	31 ± 1	32 ± 1	27 ± 1	28	28 ± 1	32 ± 1	28 ± 2	29 ± 1	33 ± 2	36 ± 1	32 ± 2	37 ± 2	32 ± 2	33 ± 1
*S. capitis* subsp*. capitis* ATCC35661	32 ± 3	35 ± 2	24 ± 1	31 ± 1	29 ± 1	31 ± 1	31 ± 1	36 ± 1	29 ± 1	32 ± 2	27 ± 1	33 ± 1	30 ± 1	38 ± 2	32 ± 2	37 ± 1	33 ± 2	37 ± 2	31 ± 2	36 ± 2
*S. intermedius* ATCC29663	38 ± 2	42 ± 2	29 ± 1	35 ± 2	30 ± 3	32 ± 2	35 ± 1	37 ± 1	30 ± 2	31 ± 3	27 ± 1	29 ± 1	28 ± 1	31 ± 1	31 ± 1	34 ± 1	33 ± 1	36 ± 1	28 ± 1	32 ± 2
*S. lentus* ATCC700403	24 ± 1	30 ± 3	25 ± 1	27 ± 1	22 ± 2	23 ± 1	34	39 ± 2	28 ± 2	27 ± 3	23 ± 3	25 ± 2	30 ± 1	30 ± 3	33 ± 1	35 ± 2	27 ± 2	31 ± 1	26 ± 2	28 ± 3
*S. lugdunensis* ATCC49576	12 ± 1	15 ± 1	29 ± 1	33 ± 3	29 ± 2	33 ± 2	35 ± 2	38 ± 2	31 ± 3	34 ± 3	24 ± 2	30 ± 2	28 ± 2	29 ± 2	30 ± 1	33 ± 1	33 ± 1	37 ± 1	28 ± 2	30 ± 1
*S. simulans* ATCC27851	19 ± 3	21 ± 3	31 ± 1	35 ± 1	31 ± 1	36 ± 3	36 ± 3	37 ± 2	36	37 ± 1	29 ± 2	31 ± 3	29 ± 1	33 ± 3	32 ± 1	36 ± 2	12	16 ± 1	28 ± 2	27 ± 1
*S. galinarium* ATCC700401	9	13 ± 1	25 ± 1	33 ± 1	21 ± 1	27 ± 1	20 ± 1	27 ± 1	27 ± 1	30 ± 0	24 ± 1	30 ± 2	26 ± 1	31 ± 1	29 ± 1	34 ± 2	9 ± 1	11 ± 1	27 ± 1	27 ± 1
*S. sciuri* ATCC29060	24 ± 1	27 ± 2	24 ± 1	27	24 ± 1	26 ± 1	28	32 ± 2	26 ± 1	26 ± 1	22 ± 1	24 ± 1	26 ± 1	29 ± 1	30 ± 1	33 ± 1	27 ± 1	31 ± 1	23 ± 1	23 ± 1
*S. xylosus* ATCC700404	9 ± 2	12 ± 1	0	0	0	0	37 ± 1	39 ± 1	30 ± 1	30 ± 2	19 ± 1	20 ± 1	28 ± 2	30 ± 2	32 ± 1	35 ± 1	14 ± 1	16 ± 1	32 ± 2	32 ± 2
average	22 ± 1	26 ± 1	23 ± 1	28 ± 1	23 ± 1	27 ± 1	31 ± 1	36 ± 2	29 ± 1	31 ± 1	25 ± 1	29 ± 1	28 ± 1	32 ± 2	32 ± 2	37 ± 1	23 ± 1	27 ± 1	28 ± 1	31 ± 1

Inhibition growth enhancement of *S. capitis* subsp*. capitis* ATCC35661 from 3 to 8 mm after addition of berberine to the MHA medium was observed. The most noticeable differences in size of the growth inhibition zone were found in the presence of E (increase from 24 to 31 mm), C (increase from 30 to 38 mm) and TOB (increase from 27 to 33 mm). These antibiotics showed a growth inhibition zone increase from 6 to 8 mm.

The effect of the interaction of berberine and antibiotics on *S. galinarium* ATCC700401 strain was expressed as the increase of inhibition growth zone by 3-8 mm. The biggest differences in the growth inhibition zones sizes were observed in case of E (increase from 25 to 33 mm), FOX (increase from 20 to 27 mm), TOB (increase from 24 to 30 mm) and DA (increase from 21 to 27 mm). For these antibiotics we noticed an increase of the inhibition zone ranging from 6 to 8 mm.

The increase of growth inhibition zones for *S. hominis* subsp*. hominis* ATCC27844 strain were between 1 to 11 mm. The biggest differences in inhibition zone growth were noted for LIN (increase from 34 to 45 mm), FOX (increase from 32 to 41 mm) and E (increase from 34 to 40 mm). For these antibiotics the most significant growth inhibition zones were within the range from 6 to 11 mm.

An increase of the growth inhibition zone within the range from 1 to 6 mm was observed for *S. lugdunensis* ATCC49576 strain. The greatest differences in growth inhibition zones were observed for TOB (increase from 24 to 30 mm), for which the increase of the growth inhibition zone was 6 mm.

For other investigated strains increases of growth inhibition zone of more than 5 mm were observed for FOX and *S. epidermidis* ATCC35983 (increase from 12 to 18 mm) and for P and *S. lentus* ATCC700403 (increase from 24 to 30 mm).

Our analysis of the influence of berberine on the antimicrobial action of the selected antibiotics, revealed an increase of the growth inhibition zone in the presence of berberine at a concentration corresponding to ¼ MIC for all examined strains. The most susceptible strains towards berberine were *S. haemolyticus* ATCC29970, *S. epidermidis* ATCC 12228, *S. capitis* subsp*. capitis* ATCC35661, *S. galinarium* ATCC700401, *S. hominis* subsp. *hominis* ATCC27844, *S. intermedius* ATCC29663, and also *S. lugdunensis* ATCC49576. Assessing the other investigated strains, such as *S. sciuri* ATCC29060, *S. epidermidis* ATCC35983, *S. saprophyticus* ATCC15303, *S. simulans* ATCC27851, *S. xylosus* ATCC700404, *S. warneri* ATCC49454 and *S. lentus* ATCC700403, the enhancement of growth inhibition following addition of berberine to MHA was observed only to a minor extent, up to 5 mm. The weakest interactions between berberine and anti-staphylococcal drugs was found for *S. xylous* ATCC70040, *S. warneri* ATCC49454, and *S. lentus* ATCC700403. 

The most significant synergistic effect was noticed for berberine in combination with LIN, FOX and E (Table 4). For all tested strains the combination of berberine and STX resulted in the appearance of a double growth inhibition zone along with a clear reduction of the microorganisms within the first inhibitory zone.

Berberine is an isoquinoline-type alkaloid isolated from many herbs. It has been reported that berberine shows relatively weak antimicrobial activity against Gram-negative bacteria and is more effective against Gram-positive ones [7,13]. The empirical multidrug antimicrobial therapy is usually applied to expand the antibacterial spectrum and reduce the selection of drug resistant mutants. In addition, combinations of different antimicrobial agents that exhibit synergy or partial synergy, may augment the antimicrobial effect in patients with persistent infections at lower concentrations. The application of antibiotics supported by bioactive substances originating from natural products seems to be a promising and efficient therapy for such infections. This can suggest a potential role of berberine as a supportive compound which may play a significant role in the reduction of adverse effects which are frequently caused by common antibiotics [14].

**Table 4:** Alteration of inhibition growth zone investigated antibiotics in combination with berberine for CoNS strains. Pearson Correlation *p* = 0.97.

	Antibiotic(Inhibition Growth Zone in mm ± SD)	Antibiotic with Berberine(Inhibition Growth Zone in mm ± SD)	Pearson Correlation
LIN	32 ± 2	37 ± 1	r = 0.62
FOX	31 ± 1	36 ± 2	r = 0.86
E	23 ± 1	28 ± 1	r = 0.97
P	22 ± 1	26 ± 1	r = 0.93
DA	23 ± 1	27 ± 1	r = 0.97
TOB	25 ± 1	29 ± 1	r = 0.77
C	28 ± 1	32 ± 2	r = 0.42
TE	23 ± 1	27 ± 1	r = 0.94
CIP	29 ± 1	31 ± 1	r = 0.73
STX	28 ± 1	31 ± 1	r = 0.34

The majority of research focusing on the antibacterial effects of berberine are related to its influence on *S. aureus* strains [9,15,16], and the combined synergistic effect of berberine along with the selected antibiotics towards *S. aureus* and *S. epidermidis* strains [10,13,17,18]. According to previous reports, both *S. aureus* strains and CoNS strains were found to show a highly variable susceptibility to antibiotics and natural products [19,20].

To the best our knowledge the presented work seems to be a pioneering study focused on the biological effect of berberine alone and in combination with the selected antibiotics on coagulase-negative staphylococci other than *S. epidermidis*. A study of the anti-staphylococcal activity of berberine on MRSA strains, carried out by Yu *et al*. [13], indicated a significantly higher susceptibility of these strains to berberine, compared to the CoNS strains included in our study, with MIC values ranging from 32 µg/mL to 128 µg/mL. Similar results were presented by Zuo *et al.* [17] who assessed MIC values for berberine and for 10 MRSA strains. In our study the MIC of berberine chloride against 14 CoNS strains ranged widely from 16 to more than 512 µg/mL. These MIC values are compared with data presented by Yu *et al.* and Zuo *et al.* which suggests that some CoNS strains may show higher resistance against berberine derivatives than MRSA strains.

In our study, the MIC values for biofilm-forming *S. epidermidis* were four times higher than for a non-biofilm forming strain (ATCC12228). A similar effect was also observed by Wang *et al.* [21] who performed a comparative investigation of the use of berberine using three reference *S. epidermidis* strains (two biofilm-forming and one non-biofilm forming) in a microbiological assessment of berberine activity on *S epidermidis* strains. The MIC values were 256 µg/mL, 128 µg/mL, and 64 µg/mL. These studies indicate a preventative role of berberine on the biofilm formation ability of *S. epidermidis*.

There are very few reports addressing the combined effects of active, natural substances such as baicalein and ciprofloxacin [22], ethanol extract of propolis and anti-staphylococcal drugs [20], flavones and β-lactam antibiotics [23], berberine and ampicillin or oxacillin [13] berberine and ampicillin, azithromycin, cefazolin or levofloxacin [17] towards *S. aureus* strains. Reports about the synergistic effects of different compounds on CoNS are rare, however they indicate the possibility of augmenting the antibacterial effect of commonly used antibiotics by adding certain natural compounds.

The results obtained suggest that berberine possesses substantial antimicrobial activity toward CoNS, and indicate that the combination of berberine with oxazolidiones (LIN), β-lactams (FOX), macrolides (E) and another antibiotics results in a synergistic reaction. The interaction of berberine with different antimicrobial agents might be attributed to a blockage of different bacterial resistance mechanisms, including the bacterial efflux pump inhibitory effect of berberine compounds [24,25]. A model of the synergistic mechanism of berberine and antibiotics is hypothetically based on blocking the NorA pump and potentiating the antibiotics’ action. Berberine, which accumulates in the cell, driven by the membrane potential may prevent the NorA pump from extruding some antibiotics [26,27]. According to previous reports by Jin *et al*. the mechanisms for the bactericidal effect of berberine may include inhibition of DNA replication, RNA transcription, and protein biosynthesis, influence or inhibition of enzyme activities, and destruction of the bacterial cell surface structure resulting in Ca^2+^ and K^+^ release from cells [9]. This synergistic effect of antibiotics along with berberine may have potential practical application in a clinical environment as a efficient measure for eradicating infectious CoNS strains, in cases requiring a non-standard pharmacological approach.

## 3. Experimental

### 3.1. Bacterial Strains, Media and Reagents

The antibacterial activity of berberine was assessed against fourteen CoNS standard strains: *S. epidermidis* ATCC12228, *S. epidermidis* ATCC35983 (biofilm positive), *S. haemolyticus* ATCC29970, *S. hominis* subsp. *hominis* ATCC27844, *S. warneri* ATCC49454, *S. saprophyticus* ATCC15303, *S. capitis* subsp. *capitis* ATCC35661, *S. intermedius* ATCC29663, *S. lentus* ATCC700403, *S. lugdunensis* ATCC49576, *S. simulans* ATCC27851, *S. galinarium* ATCC700401, *S. sciuri* ATCC29060 and *S. xylosus* ATCC700404. Bacterial strains were stored for further analyses in TSB (Tryptic Soy Broth) medium with 20% of glycerol at −80 °C and used as required. Tryptic soy broth (TSB) and Mueller-Hinton Agar (MHA) were obtained from (BTL, Łódź, Poland). The berberine chloride (C_20_H_18_ClNO_4_, molecular weight 371.81) used in this study was obtained from Sigma Chemical Co. (St. Louis, MO, USA) Berberine was dissolved in deionized water and filtered through a 0.22 µm Millipore filter (Sartorius Co. Bohemia, NY, USA) before use. 

### 3.2. Microdilution Method

The MICs of berberine were determined by a microtitre broth dilution method. Growth inhibition assays were performed in sterile 96-well plates (FL Medical, Torreglia, Italy) in a final volume of 200 μL [28,29]. The cell concentrations were estimated from the optical densities at 600 nm wavelength with the formula CFU/mL = *A*_600_ (3.8 × 10^8^), where CFU was the number of colony-forming units. One hundred microliters of mid-logarithmic-phase bacterial cultures (5 × 10^5^ CFU/mL) in TSB was added to 100 μL of serially diluted berberine (0.5, 1, 2, 4, 8, 16, 32, 64, 128, 256, and 512 µg/mL). Wells containing TSB with bacterial inoculum only served as the bacterial growth control (GC). Additional controls included TSB alone (medium sterility control), TSB with different concentrations of berberine and bacterial inoculum. All samples were prepared in triplicates. Microplates were incubated at 37 °C for 2, 6, 12 and 24 h, and the bacterial cell growth was assessed by measuring the optical density of cultures at 600 nm wavelength with a Multiskan EX microplate reader (Thermo Electron Corp., Vantoa, Finland) [30,31]. MICs were defined as the lowest berberine concentration that yielded no visible growth after 24 h of incubation [28,29].

### 3.3. Disk Diffusion Method

All isolates were tested for antimicrobial susceptibility by the disk diffusion method-based analysis, using MHA and commercially available disks containing an antimicrobial agent according to the EUCAST recommendations [32]. For disk diffusion testing, 90 mm plates with the agar medium were inoculated by swabbing the agar with a swab soaked in a bacterial suspension of 1 × 10^8^ cells/mL. Disks (EMAPOL, Gdańsk, Poland) containing penicillin (P) 1 IU, erythromycin (E) 15 µg, clindamycin (DA) 2 µg, cefoxitin (FOX) 30 µg, ciprofloxacin (CIP) 5 µg, tobramycin (TOB) 10 µg, chloramphenicol (C) 30 µg, linezolid (LIN) 10 µg, tetracycline (TE) 30 µg or trimethoprim with sulfamethoxazole (SXT) 1.25 + 23.75 µg were used for the analysis of antimicrobial susceptibility.

The combined effect of antibiotics and berberine was studied using plates with MHA plus one-fourth of the MIC of berberine, which was considered as a sub-inhibitory concentration [33,34]. Disks were placed onto an agar surface and gently pressed to ensure contact using sterile forceps. Plates were incubated at 35 °C for 20 h in air. The susceptibility testing of each antibiotic for each isolate and the reference strains was performed in triplicate. After the incubation period diameters of the growth inhibition zones (in mm) were measured for each strain, and the mean values were calculated.

### 3.4. Statistical Analyses

To determine the percentage of the variation attributable to the factors such as bacterial strains, time, and concentrations the results concerning the bacterial growth were analyzed by a three-way analysis of variance (ANOVA). The results from synergism assay were submitted to the Wilcoxon Signed-Rank Test and Mann Whitney U Test comparing the values (mm) of the inhibitory zone in the disk diffusion method. The statistical analyses were performed using the Statistica 10.0 PL software package, assuming the statistical significance level of *p* ≤ 0.05.

## 4. Conclusions

The antimicrobial effect of berberine chloride in combination with various anti-staphylococcal drugs on reference CoNS strains varied greatly depending on the bacterial strain and drug used. The most significant synergistic effects towards CoNS strains were noted when berberine was combined with linezolid, cefoxitin and erythromycin. Our data showed a synergy of berberine and antibiotics in the antimicrobial action against some reference CoNS strains *in vitro*, what seems to suggest a potential role of berberine as a compound capable of augmenting the action of common antibiotics *in vivo*. Future work is necessary to precisely assess the effects of berberine in the treatment of CoNS infections, which may result in the development of efficient new therapeutic strategies for persistent staphylococcal infections.
